# Machine learning-based classification analysis of knowledge worker mental stress

**DOI:** 10.3389/fpubh.2023.1302794

**Published:** 2023-11-07

**Authors:** Hyunsuk Kim, Minjung Kim, Kyounghyun Park, Jungsook Kim, Daesub Yoon, Woojin Kim, Cheong Hee Park

**Affiliations:** ^1^Mobility UX Research Section, Electronics and Telecommunications Research Institute, Daejeon, Republic of Korea; ^2^Division of Computer Convergence, Chungnam National University, Daejeon, Republic of Korea

**Keywords:** heart rate, machine learning, mental stress, knowledge worker, photoplethysmography, pulse rate variability

## Abstract

The aim of this study is to analyze the performance of classifying stress and non-stress by measuring biosignal data using a wearable watch without interfering with work activities at work. An experiment is designed where participants wear a Galaxy Watch3 to measure HR and photoplethysmography data while performing stress-inducing and relaxation tasks. The classification model was constructed using k-NN, SVM, DT, LR, RF, and MLP classifiers. The performance of each classifier was evaluated using LOSO-CV as a verification method. When the top 9 features, including the average and minimum value of HR, average of NNI, SDNN, vLF, HF, LF, LF/HF ratio, and total power, were used in the classification model, it showed the best performance with an accuracy of 0.817 and an F1 score of 0.801. This study also finds that it is necessary to measure physiological data for more than 2 or 3 min to accurately distinguish stress states.

## 1. Introduction

Low-moderate levels of perceived stress have been shown to be associated with increased Working Memory (WM)-related neural activation, resulting in more optimal WM behavioral performance (1). However, higher stress scores are associated significantly with lower productivity scores (2). Stress can affect health directly through autonomic and neuroendocrine responses, but it can also affect health indirectly through changes in health behaviors (3). Mental stress in workers can reduce the quality of labor and increase a nation’s economic and industrial losses due to high medical costs and related insurance payments.

Recent studies have aimed to objectively quantify mental stress by analyzing physiological responses to stress using wearable sensors (4–6). Lee et al. (4) measured Electrocardiogram (ECG) and Electroencephalogram (EEG) data while the participants played money games, and they analyzed the effects of stress on human physiological response. The ECG sensors were attached based on the bipolar limb leads and 14 EEG channels were attached to the scalps of the participants. In a study by Acerbi et al. (5), ECG information was collected using a wearable Bluetooth chest belt, and Galvanic Skin Responses (GSR) were collected using a finger-type GSR sensor. Their analyzes of the ECG and GSR data highlighted significant differences between stressed and non-stressed individuals. In a study conducted by Chalmers et al., Heart Rate (HR) was measured using a wearable Fitbit Versa 2 device on the nondominant wrist, and HR Variability (HRV) was measured using a three-lead ECG on the chest. In the stress state, the HR and the Low-Frequency (LF) and High-Frequency (HF) increase significantly (6). However, it is disruptive for workers to wear these devices and measure their biosignal information at work.

In addition, researches are being conducted to collect data using wearable watches and then apply machine learning techniques to measure mental stress (7–9). Arsalan and Majid (7) used electroencephalography, GSR, and Photoplethysmography (PPG) signal data acquired during the resting state and public speaking activities to classify stressed and non-stressed groups. The classification was performed using five different classifiers. Dalmeida and Masala (8) collected HR from four Apple Watch users during a break while listening to relaxing music and after an 8-h workday. After extracting and normalizing HRV features from HR, they split the training and testing datasets 80:20 and used the Multilayer Perceptron (MLP) classifier. Can et al. (9) collected heart activity, skin conductance and accelerometer signals using Empatica E4 and Samsung Gear from algorithm programming competition participants. They discriminated contest stress, relatively higher cognitive load (lecture) and relaxed time activities by using different machine learning methods.

However, to develop a system that can monitor and identify the current mental stress of knowledge workers at work, it is necessary to measure and analyze physiological data by simulating their work and rest behaviors. Additionally, noninvasive methods that can quickly measure biosignals to classify and predict mental stress without disrupting work are required. Thus, we set the following research questions and designed an experiment to measure the mental stress state of knowledge workers by performing stressful tasks and relax tasks.

• Is it possible to classify stressed and non-stressed states using biosignals data measured by a wearable watch?

• For the prediction of stressed and non-stressed states, how long is it appropriate to measure biosignal data with a wearable watch?

## 2. Experimental environments for data collection

### 2.1. Experiment environment

The experiment in this study were approved by the Korean Public Institutional Bioethics Committee (http://public.irb.or.kr/; approval number: P01-202109-13-002). The 80 participants were involved in the experiment and data from 13 subjects were excluded from the analysis for reasons including device malfunction, missing some data, and abnormal data collection due to Bluetooth communication errors. The 67 participants used in the analysis were 39 men (58%) and 28 women (42%), with an average age of 36.5 years (standard deviation 8.6 years).

**Figure 1:**
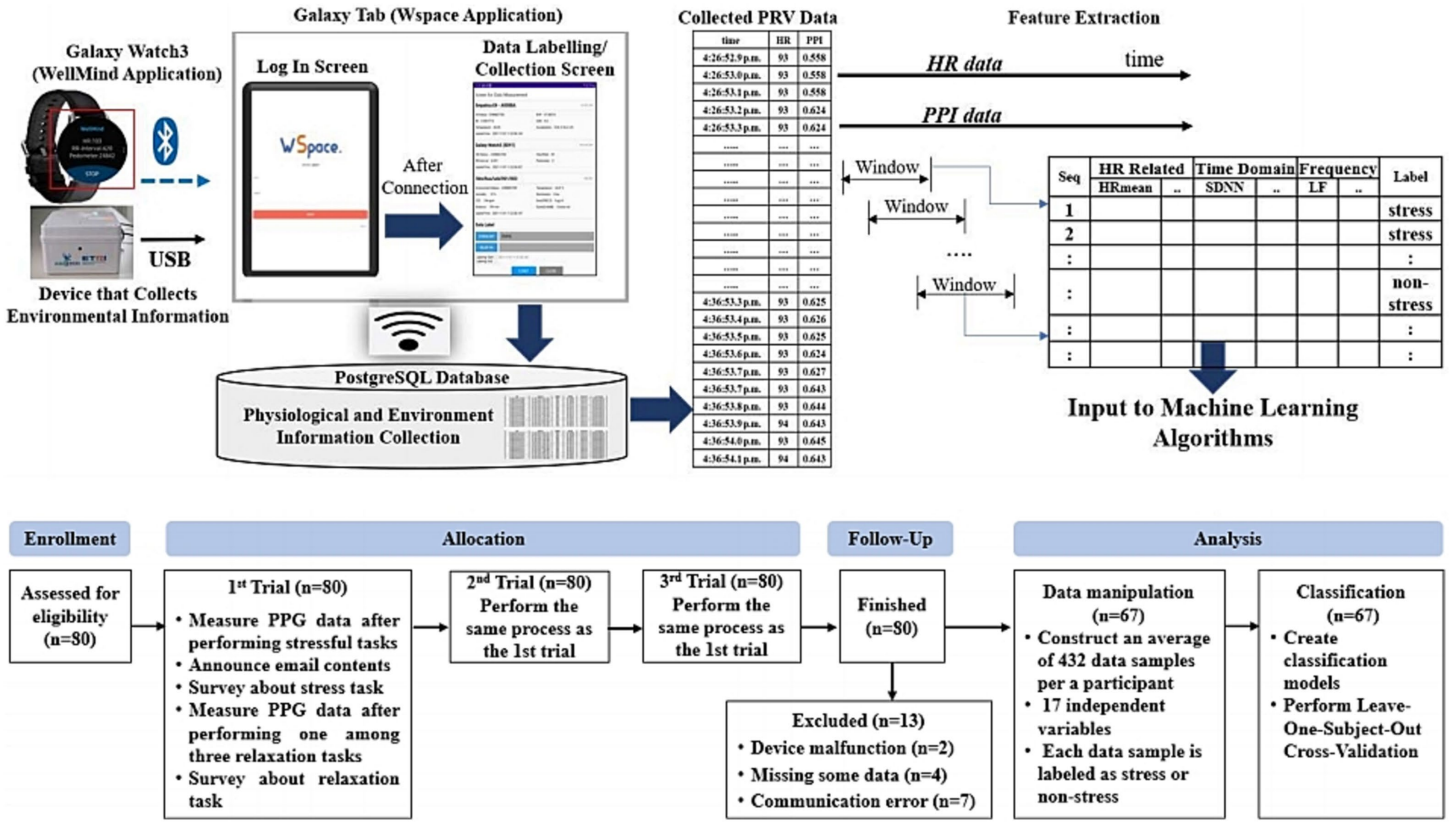
Experiment design and data feature extraction.

The top left of Figure 1 represents the data collection environment. We developed the WellMind Application (App) and installed on the Samsung Watch3 to collect the HR and peak to peak interval (PPI) data from the Watch3 and to transmit the data to the Galaxy Tablet. We developed an application called WellMind Space (WSpace), installed it on a tablet, connected the Watch3 and tablet via Bluetooth, and collected data using the app. The WSpace possesses a labeling function that permits the annotation of stressful and relaxing task data as stress and non-stress labels, respectively. All data were stored on a computer installed with PostgreSQL (10).

### 2.2. Experimental procedure

The experimental procedure was as follows.

*Preparation*: The participants completed the consent form and profile questionnaire and then placed the Watch3 on their wrists. The operator established a Bluetooth connection between the Watch3 and the WSpace.

*Stress task*: The participants followed the operator’s instructions and performed a stress task for 5 min, that is, the operator sent the participants three emails which asked to search for information on the specific topics at 1 min intervals and each replied separately to three emails. This stress-inducing task was chosen following (11), where email writing was used as a stress-inducing task. In addition, to keep the participants’ stress level during the physiological data measurement, they were asked to memorize contents of the email for later presentation.

*Measurement of PPG data after completing the stress task*: The operator measured the participants’ HR and PPI data using the Watch3.

*Announce email contents*: Participants had to announce the contents of the email; this was done to keep participants stress state after the stress task before the PPG data measurements.

*Survey about stress task*: After announcing the email content, the participants completed a survey on their experiences with stress.

*Relaxation task*: Participants then performed one of three relaxation tasks: closing their eyes, stretching, or using a massager. The participants were divided into three groups to account for counterbalancing, and each group performed the relaxation tasks in a different order.

*Measurement of PPG data after completing the relaxation task*: The operator measured the participants’ HR and PPI data.

*Survey about relaxation task*: After performing the relaxation task, the participants completed a questionnaire about their relaxation task.

Participants repeated the above procedure three times. The bottom of Figure 1 represents the experiment procedure diagram.

## 3. Data manipulation

### 3.1. Photoplethysmography

PPG sensor uses a photodetector to measure the intensity of light reflected from the tissue, and changes in blood volume can be measured depending on the amount of light detected. Similar to ECG, PPG exhibits stable cardiac and respiratory activity. PPI defined as the time interval between successive peaks of the PPG waveform, can be utilized to derive the Pulse Rate Variability (PRV), which shares similarities with the ECG-derived HRV (12).

Because mental stress affects the Autonomic Nervous System (ANS), PRV is a means to observe ANS responses indirectly. Therefore, studies are being conducted to classify and predict the presence or absence of stress state using PPG signals (7, 13). HRV data can be used for stress detection by analyzing the time- and frequency-domain features (7, 13, 14). In particular, the Standard Deviation of Normal-to-Normal intervals (SDNN) and Root Mean Square of Successive Differences between normal heartbeats (RMSSD) which are related to the interval between consecutive heartbeats (the interbeat interval) and LF/HF in the frequency domain appear to be the primary factors that differentiate stress states. These features can also be used when analyzing PRV (12).

### 3.2. Feature variables

To analyze the mental stress of knowledge workers during working hours, it is necessary to acquire biosignals from these workers without disturbing them. Therefore, wrist-worn devices are more user-friendly in daily life than chest-worn devices. The Watch3, which integrates a PPG sensor to measure light intensity changes in the microvascular tissue and derive HR and PPI information, is worn on the wrist and offers a convenient and noninvasive approach for HR and PPI measurements (15). Hence, this study employed a Watch3 to collect these data.

The top right of Figure 1 illustrates the process of extracting features from HR and PPI data sequences. After completing each task, participants had 5 to 7 minutes of physiological data measurements. Moving a 3-min window forward with a shift size of 10 s in the HR and PPI data sequences collected from each participant, a total of 17 independent features were extracted from the data within each window to form a data sample. The minimum, mean, median, and maximum values were calculated from a window in the HR data sequence. Time-domain and frequency-domain features were calculated from a window in the PPI data sequence. Time-domain features include the average NN Intervals (NNI), RMSSD, SDNN, Standard Deviation of Differences between adjacent NN intervals (SDSD), Percentage of successive NN intervals that differ by more than 50 ms (PNN50), and PNN20 values. Frequency domain features include LF, HF, LF/HF ratio, LF power in normalized units (LFnu), HF power in normalized units (HFnu), total power, and very Low Frequency (vLF).

The label “stress” was assigned to data samples which were constructed from physiological data measured when performing the stress task, and the label “non-stress” was assigned to data samples obtained from the relaxation task. Since one participant performs 6 tasks and the measurements were made over 5 min for each task, an average of 432 data samples per a participant can be obtained. Physiological data varies depending on each subject’s personal health status. Subsequently, min–max normalization was applied to each feature of each participant to generate the final data features for analysis. The collection of all data samples from all participants was used as an input to machine learning algorithms for binary classification of stress and non-stress.

## 4. Classification results

### 4.1. Classification analysis

In this study, k-Nearest Neighbor (k-NN), Support Vector Machine (SVM), MLP, Decision Tree (DT), Random Forest (RF), and Logistic Regression (LR) classifiers of the scikit-learn library was used (16). To achieve the highest performing classification model, hyperparameter tuning was performed using GridsearchCV function for each algorithm used. To evaluate the classifiers, Leave-One-Subject-Out Cross-Validation (LOSO-CV) were performed. In LOSO-CV, from the 67 participants, the data for 66 people were used as the training set, and the data from one participant was used as the test set. This process was repeated 67 times to measure the performance and to calculate the average to determine the overall performance.

The classification models were evaluated using the accuracy, precision, recall, and F1 scores as evaluation measures, as shown in Eqs. 1–4 (17), based on the confusion matrix. True Positive (TP) is the number of data samples predicted to be positive when belonging to the positive class. False Positive (FP) is the number of data samples predicted to be positive when belonging to the negative class. True Negative (TN) and False Negative (FN) are defined similarly. Matthews Correlation Coefficient (MCC) as expressed in Eq. 5 can also be used to evaluate the performance of the classification model (18).


(1) $$\mathrm{accuracy}=\frac{TP+TN}{TP+FN+FP+TN}$$

(2) $$\mathrm{precision}=\frac{TP}{TP+FP}$$

(3) $$\mathrm{recall}=\frac{TP}{TP+FN}$$

(4) $$F1-\mathrm{score}=2\times \frac{\mathrm{Precision}\times \mathrm{Recall}}{\mathrm{Precision}+\mathrm{Recall}}$$

(5) $$MCC=\frac{\left(TP*TN\right)-\left(FP*FN\right)}{\sqrt{\left(TP+FN\right)\left(TN+FP\right)\left(TP+FP\right)\left(TN+FN\right)}}$$

In this study, the positive corresponds to the stress state and the negative corresponds to the normal state. To accurately predict the stress state, it is important to optimize the performance measures of TP and TN and minimize the occurrence of FP and FN. In particular, a high TP rate (correct identification of stressed cases) and a low FN rate (correct identification of unstressed cases) are crucial. It is necessary to find a model with a high-recall value to effectively predict stressed knowledge workers and guide them to take breaks. High F1 scores indicate that the corresponding classification model effectively predicts stressed workers.

This study constructs six machine-learning models and conducted a classification analysis to determine whether mental health state was categorized as either stress or non-stress.

Table 1 lists the results of the classification analysis using the LOSO-CV for the data generated using a 3-min window. The results showed that the LR classifiers achieved the best performance with accuracy of 0.814, precision of 0.843, recall of 0.805, F1 score of 0.796, and MCC of 0.643. The k-NN classifier achieved the lowest performance with an accuracy of 0.719 and an F1 score of 0.692.

**Table 1:** Classification analysis using leave-one-subject out CV for data generated using a 3-min window.

Classifier	Accuracy	Precision	Recall	F1	MCC
k-NN	0.719	0.729	0.700	0.692	0.426
SVM	0.743	0.766	0.730	0.723	0.491
MLP	0.741	0.760	0.725	0.718	0.482
DT	0.756	0.787	0.739	0.730	0.519
RF	0.788	0.821	0.770	0.766	0.585
LR^a^	0.814	0.843	0.805	0.796^a^	0.643

a Highest F1 score.

### 4.2. Window size

Further analysis was conducted to determine the optimal window size required for measuring physiological data to predict the stress experienced by knowledge workers during working hours. In the analysis by LOSO-CV, the LR classifier was used as the prediction model owing to its best performance, as shown in Table 2, and various window sizes ranging from 30 s to 300 s (with 30-s intervals) were tested. This analysis aimed to identify the most appropriate time for measuring physiological data during working hours to accurately predict the stress status of workers.

**Table 2:** Results of logistic regression classification analysis after changing the window size.

Size	Accuracy	Precision	Recall	F1	MCC
30 s	0.762	0.792	0.758	0.747	0.545
60 s	0.775	0.806	0.770	0.761	0.572
90 s	0.795	0.825	0.791	0.782	0.612
120 s	0.801	0.832	0.796	0.787	0.624
150 s^a^	0.816	0.843	0.807	0.800^a^	0.646
180 s	0.814	0.843	0.805	0.796	0.643
210 s	0.805	0.842	0.792	0.784	0.628
240 s	0.808	0.846	0.792	0.784	0.630
270 s	0.815	0.848	0.791	0.787	0.632
300 s	0.826	0.862	0.793	0.792	0.646

a Highest F1 score.

The classification accuracy significantly improved when the window size was greater than 2 min. The highest performance was achieved when the window size was set to 150, with an accuracy of 0.816, precision of 0.843, recall of 0.807, F1 score of 0.8, and an MCC value of 0.646. It can be suggested that measuring physiological data for at least 2–3 min is necessary to accurately distinguish between stressed and non-stressed states in knowledge workers.

### 4.3. Feature selection

Performance improvements in classification models typically depend on the selection of a suitable set of features. Gioia et al. (19) used a feature selection strategy based on Recursive Feature Elimination (RFE).

**Figure 2:**
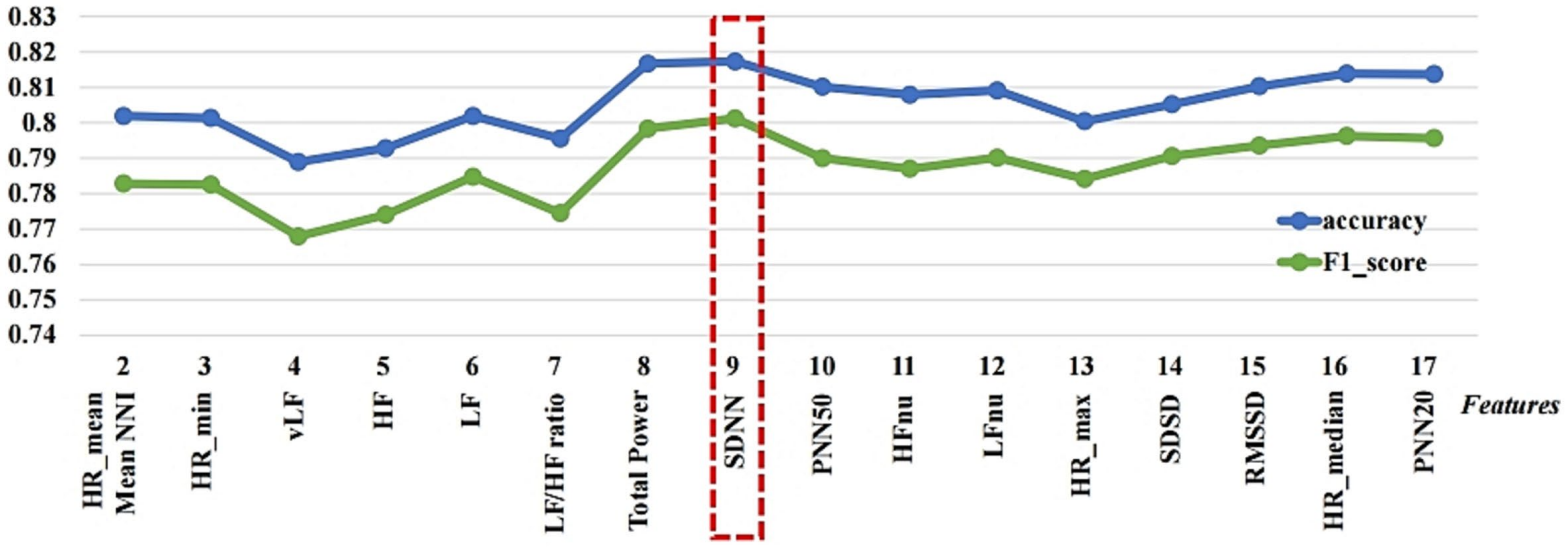
Comparison of accuracy and F1-scores when feature subsets are used based on feature ranking.

This study employs the LR-RFE model to determine how performance varies depending on the features utilized. Figure 2 compares the performance of LOSO-CV using the selected features after the feature rank is determined by applying RFE with LR to the entire dataset. The features are displayed on the x-axis based on rank, and the accuracy and F1 score are measured by adding the features in the top rank individually. The best performance was obtained with an accuracy value of 0.817 and an F1 score of 0.801 when nine top-ranked features were used, including 2 HR-related features, HR_mean and HR_min; two time-domain features, Mean_NNI and SDNN; and five frequency-domain features, vLF, HF, LF, LF/HF ratio, and Total Power.

## 5. Discussion and conclusions

Since mental stress can reduce the quality of work and worsen health conditions, the technology needed for a mental health management system that monitors the mental stress of knowledge workers in the workplace must continue to be researched. In this study, HR and PPI data were measured using the Galaxy Watch3 rather than a chest-worn ECG device. The classification model was constructed using k-NN, SVM, DT, LR, RF, and MLP classifiers. The performance of each classifier was evaluated using LOSO-CV as a verification method.

To determine the optimal duration for measuring biosignals to classify and predict the mental stress, the HR and PRV data features were calculated using varying window sizes. The window size was varied from 30 to 300 s. The best performance was achieved with an accuracy of 0.816, precision of 0.843, recall of 0.807, F1 score of 0.8, and MCC of 0.646, using an LR classifier with 17 features extracted by setting the window size to 150. The results of this study show that it is possible to analyze mental stress using PPG data obtained over a sufficiently short period of time of 2 to 3 min that does not interfere with work activities at work.

Additionally, the LR-RFE model was utilized to investigate how performance changes depending on the type of features used. The best performance exhibited an accuracy value of 0.817 and an F1 score of 0.801 when the nine top-ranked features were used. The feature selection results can be used to achieve a classification model suitable for highest performance.

To develop a system that can measure mental stress during working hours and guide rest in the event of stress, it is necessary to obtain biosignals without disturbing workers. In our study, HR and PPI data were collected using a Watch3 to noninvasively measure biosignals. However, it should be noted that PPG signals have a limitation in that they are sensitive to motion artifacts caused by hand movements (20). Recently, studies on HR measurement methods based on remote PPG detection using deep learning-based facial videos have also been implemented (21, 22). In order to minimize worker inconvenience and simultaneously improve prediction performance, research should be conducted on stress analysis through the combination of facial images and biosignal information through wearable watches. Additionally, we plan to conduct research on the development of stress monitoring and intervention apps that incorporate these technologies.

## Data availability statement

The datasets presented in this article are not readily available because the data analyzed in this study is subject to the following licenses/restrictions: the data is the property of the Mobility UX Research Section, Electronics and Telecommunications Research Institute, Republic of Korea. Requests to access the datasets should be directed to HK, hyskim@etri.re.kr.

## Author contributions

HK: Formal analysis, Funding acquisition, Investigation, Methodology, Project administration, Software, Supervision, Visualization, Writing – original draft, Writing – review & editing. MK: Conceptualization, Resources, Writing – original draft. KP: Conceptualization, Resources, Writing – original draft. JK: Conceptualization, Resources, Writing – original draft. DY: Funding acquisition, Project administration, Writing – original draft. WK: Funding acquisition, Project administration, Writing – original draft. CP: Methodology, Visualization, Writing – review & editing.
